# Accelerometer-Based Step Counts May Be Confounded by Repetitive Motor Behaviors in Adults With Autism Spectrum Disorder

**DOI:** 10.2196/110988

**Published:** 2026-10-30

**Authors:** Muhammad Anas Malik, Tayeba Fatima

**Affiliations:** 1 Ameer-ud-din Medical College, 6-Abdur Rehman Chughtai Road (Birdwood Road), near Jail Road, Shadman, Lahore, Punjab, Pakistan, 92 3095722589; 2 Avicenna Medical College, Lahore, Punjab, Pakistan

**Keywords:** autism spectrum disorder, physical activity, co-participation, family support, autism in adulthood, telehealth, wearable devices, physical function, remote assessment, health disparities, minority health, adult autism, protocol study, cross-sectional design, patient-reported outcomes, motor impairments, rehabilitation, digital health

We read with interest the recent pilot study by Nunez-Gaunaurd et al [1] involving remote assessment of physical activity and physical function in adults with autism spectrum disorder (ASD). The integration of accelerometer-based step monitoring with remote performance-based and patient-reported measures represents a valuable step toward accessible, telehealth-based functional assessment in an underserved population. However, we found one measurement concern relevant to the study’s validity aims.

Consumer accelerometers such as the Fitbit Inspire 3 estimate step counts using gait-pattern algorithms calibrated primarily on people with neurotypical ambulatory movements, and even in this population, they can record false step counts, as mentioned in a recent study [2]. Adults with ASD frequently exhibit repetitive, uncontrolled motor behaviors like hand flapping, rocking, and pacing that generate rhythmic upper- and lower-limb acceleration signals that are distinct from ambulatory movements during walking and can potentially be misclassified as ambulatory steps [3]. The study by Nunez-Gaunaurd et al [1] proposes using step counts per day as a criterion indicator to establish the construct validity of Patient-Reported Outcomes Measurement Information System (PROMIS) physical function scores, so any systematic over- or undercounting of steps due to characteristic ASD movement patterns could bias this validation. This concern is distinct from the generalizability and sampling limitations the authors already mentioned in this population.

We would ask the study team whether they have planned or piloted any validation of Fitbit-derived step counts against a gold standard method, like video-coded step counts, in a subsample of participants with prominent motor stereotypes, which would help establish whether this measure can be trusted as a criterion standard in this population [4].

This is an addressable question rather than a fundamental design flaw, and clarification would strengthen confidence in the study’s future findings.

## Abbreviations

ASD: autism spectrum disorder
PROMIS: Patient-Reported Outcomes Measurement Information System

## References

[1] Nunez-Gaunaurd A, Tovin MM, Hale M, Li T, Bhat A (2026). Remote assessment of physical function and physical activity in adults with autism spectrum disorder: protocol for feasibility, validity, and reliability study. JMIR Res Protoc.

[2] Modave F, Guo Y, Bian J (2017). Mobile device accuracy for step counting across age groups. JMIR Mhealth Uhealth.

[3] Melo C, Ruano L, Jorge J (2020). Prevalence and determinants of motor stereotypies in autism spectrum disorder: a systematic review and meta-analysis. Autism.

[4] Toth LP, Park S, Springer CM, Feyerabend MD, Steeves JA, Bassett DR (2018). Video-recorded validation of wearable step counters under free-living conditions. Med Sci Sports Exerc.

